# Modulation of the biological network of lumbar spinal stenosis by Tongdu Huoxue Decoction based on clinical metabolomics

**DOI:** 10.3389/fmolb.2023.1074500

**Published:** 2023-03-21

**Authors:** Luhong Ji, Ping Huang, Qiong Wang, Xugui Li, Ying Li

**Affiliations:** ^1^ Hubei University of Chinese Medicine, Wuhan, Hubei, China; ^2^ Department of Rehabilitation Medicine, Central Theater General Hospital, Wuhan, Hubei, China; ^3^ Hubei 672 Orthopaedic Hospital of Integrated Traditional Chinese and Western Medicine, Wuhan, Hubei, China

**Keywords:** Tongdu Huoxue Decoction, lumbar spinal stenosis, metabolomics, pain, inflammation

## Abstract

**Objective:** To explore the clinical efficacy and metabolic mechanism of Tongdu Huoxue Decoction (THD) in treating lumbar spinal stenosis (LSS).

**Methods:** A total of 40 LSS patients and 20 healthy participants were recruited from January 2022 to June 2022. The patients’ pre- and post-treatment visual analogue scale (VAS) and Japanese Orthopaedic Association (JOA) scores were recorded. ELISA kits were used to assess pre- and post-treatment levels of serum Interleukin-1beta (IL-1β), Alpha tumour necrosis factor (TNF-α) and prostaglandin E2 (PGE2). Finally, the patients’ pre- and post-treatment and healthy human sera were subjected to extensively targeted metabolomics using Ultra Performance Liquid Chromatography (UPLC) to identify potential differential metabolites and metabolic pathways using multivariate statistical analysis.

**Results:** Compared to the pre-treatment (group A), the patients’ VAS scores decreased significantly (*p* < 0.05), while JOA scores increased significantly (*p* < 0.05) post-treatment (group B), indicating that THD could effectively improve the pain and lumbar spine function of LSS patients. Moreover, THD could effectively inhibit the expression of IL-1β, TNF-α and PGE2-associated inflammatory factors in serum. Regarding metabolomics, the levels of 41 differential metabolites were significantly different in the normal group (group NC) compared to group A, and those were significantly restored after treatment with THD, including chenodeoxycholic acid 3-sulfate, taurohyodeoxycholic acid, 3,5-Dihydroxy-4-methoxybenzoic acid, pinocembrin. These biomarkers are mainly involved in purine metabolism, steroid hormone biosynthesis and amino acid metabolism.

**Conclusion:** This clinical trial demonstrated that THD is effective in improving pain, lumbar spine function and serum levels of inflammation in patients with LSS. Moreover, its mechanism of action is related to the regulation of purine metabolism, steroid hormone biosynthesis and the expression of key biomarkers in the metabolic pathway of amino acid metabolism.

## 1 Introduction

Lumbar spinal stenosis (LSS) is a degenerative disease of the lumbar spine. It can lead to increased pressure in the spinal canal, causing cauda equina ischaemia or compression of the nerve roots, resulting in low back pain. The pain may be confined to the lower limbs or span multiple dermatomes, as well as impaired movement or sensation during walking, intermittent claudication and other clinical symptoms that seriously impact people’s productivity and quality of life (Deer et al., 2022). Studies have reported an 11% prevalence of symptomatic LSS in the general population, with almost half of those over 60 years experiencing symptomatic LSS. The number of people with disabilities due to LSS is expected to increase globally as the population ages rapidly (Young et al., 2020). Surgical intervention is common, but postoperative pain and disability might persist, and studies have noted that no significant benefit has been observed with surgical treatment compared to non-surgical treatment (Zaina et al., 2016). The 2nd edition of the North American Spine Surgery Society (NASS) Guidelines for the Management of Degenerative Lumbar Spinal Stenosis recommends that patients with mild to moderate lumbar spinal stenosis be considered for conservative treatment with medication, physical therapy and functional exercise (Kreiner et al., 2013). Therefore, non-surgery management is urgently needed for patients, and previous studies have shown that Chinese medicine has clear advantages in treating LSS, with the holistic concept and evidence-based treatment being its main philosophy (Zhao et al., 2019).

Tongdu Huoxue Decoction (THD) is an experienced formula for treating degenerative spinal stenosis and consists of *Cervus elaphus Linnaeus* (Lu Jiao), *Cibotium barometz* (L.), *J.Sm.* (Jinmao Gouji), *Eucommia ulmoides Oliv.* (Du Zhong), *Astragalus membranaceus* (Fisch.) *Bge.var.mongholicus* (Bge.) *Hsiao* (Huang Qi), *Angelica sinensis* (Oliv.) *Diels* (Dang Gui), *Caesalpinia sappan L.* (Su Mu), *Lycopus lucidus Turcz. var. hirtus Regel* (Ze Lan Ye), *Pheretima aspergillum* (E.Perrier) (Di Long), *Paeonia lactiflora Pall.*(Chi Shao), and *Salvia miltiorrhiza Bge.* (Dan Shen). These substances have good anti-inflammatory and analgesic effects and can effectively reduce patients’ clinical symptoms and improve the quality of life (Shu et al., 2005; Wu and Dong, 2016). THD was created by Chinese orthopaedic specialist Li Tongsheng and is mentionedin his book “*Famous Doctors’ Remedies for Experimentation*”. It has been reported that THD can improve the microcirculatory perfusion of the spinal stenosis lesion, improve local nerve root ischemia and hypoxia, accelerate the action of local inflammatory mediators and pain-causing factors, thereby reducing the patient’s back pain and improve the function of the lower limbs (Li and Li, 1991). Some studies found that THD-containing serum can slow down the degeneration of the intervertebral disc annulus by reducing the expression of NLRP3 inflammatory vesicle-mediated caspase-1 signalling pathway-related cytokines, inhibiting lipopolysaccharide/ATP-induced cell scorching, and controlling the release of inflammatory factors (Wu et al., 2021). This might be the pharmacodynamic basis for THD’s anti-inflammatory and analgesic effects. However, the effects of metabolites in treating LSS with THD are unclear. Alterations in metabolites can regulate gene transcription, and gene remodelling occurs when abnormal expression of some proteins is corrected, suggesting that changes in certain metabolic processes occur at the epigenetic level (Gao et al., 2022).

Metabolomics is capable of qualitative and quantitative analysis of metabolites in organisms. It can be used to study the changes in biomarkers and interference pathways in the body after Traditional Chinese Medicine (TCM) interventions and to scientifically interpret the efficacy of TCM interventions in diseases and their mechanisms of action (Wu et al., 2022). One study demonstrated tissue metabolism during intervertebral disc degeneration by correlating rat and human metabolomics and found that the degenerative process was associated with changes in carbohydrate utilisation patterns in the Gly-Ser-Thr metabolic axis and reduced antioxidant capacity in the *in-vitro* diagnostic environment. This process ultimately leads to the disintegration of the fibrous ring and the loss of water fixation groups (Wu et al, 2021). Moreover, metabolite changes in both serum and disc tissue of patients with disc degeneration are associated with amino acid metabolism, with increased glycine levels in both (Swank et al., 2020). This may provide ideas for treating patients with lumbar disc herniation or lumbar spinal stenosis. In addition, a broadly targeted metabolomic serum analysis using UPLC revealed that the Chinese medicine Zuojin pill was able to act as an inflammatory inhibitor, inhibiting levels of the inflammatory factors COX-2, IL-4 and IL-17, thereby regulating the combined metabolic disorders in patients with chronic non-atrophic gastritis (Ma et al., 2022). These studies offer the possibility of technical applications of THD in LSS. Therefore, we conducted a clinical trial to validate THD’s analgesic and anti-inflammatory effects in LSS and explore the underlying mechanisms using metabolomics to provide a scientific basis for clinical treatment.

## 2 Methods and design

### 2.1 Patients and methods

We recruited patients attending the Department of Spine Surgery, Hubei 672 Orthopaedic Hospital of Integrated Traditional Chinese and Western Medicine, from January 2022 to June 2022. The ethical approval was granted by the Ethics Committee of Hubei 672 Orthopaedic Hospital of Integrated Traditional Chinese and Western Medicine (672HREC20220115A).

The inclusion criteria were as follows:1) the diagnostic criteria for LSS were met, and the clinical diagnosis of LSS was determined by a combination of clinical symptoms and radiological findings of LSS on computed tomography (CT) or magnetic resonance imaging (MRI). Computed tomography or MRI shows narrowing of the lumbar spinal canal with compression of the cauda equina by thickened posterior vertebral bodies, small joints, marginal bony bulges or soft tissue structures such as the ligamentum flavum or disc herniation (Alvarez and Hardy, 1998); 2) age 18–70 years; 3) no indication for the surgery; 4) voluntary signed informed consent form. Exclusion criteria are as follows: 1) patients with other severe orthopaedic conditions; 2) those with severe medical conditions; 3) psychiatric patients; 4) pregnant or lactating women, allergic patients or those who cannot receive this treatment; 5) those who were already receiving other treatments that may affect the results of this study. The criteria for discontinuation/exclusion/withdrawal were as follows: 1) patients with severe adverse reactions during the study; 2) patients with poor compliance; 3) patients who were assessed to have difficulty tolerating the study protocol during the study. Discontinuation: In the event of withdrawal due to allergy or other adverse reactions to the study drug, the participant would be offered a specialist consultation and medication at no cost. All participants provided written informed consent and were screened by physical examination, medical history assessment and clinical laboratory tests.

Forty eligible patients were recruited to receive THD as the treatment. THD consisted of Lu Jiao 18 g, Jinmao Gouji 12 g, Du Zhong 9 g, Huang Qi 18 g, Dang Gui 9 g, Su Mu 9 g, Ze Lan Ye 9 g, Di Long 9 g, Chi Shao 9 g, and Dan Shen 18 g. All the above drugs were provided by the pharmacy of Hubei 672 Orthopaedic Hospital of Integrated Traditional Chinese and Western Medicine. Twenty general participants were used as the healthy group. The mean age of the patients was 50.83 ± 8.15 years, and the mean age of the normal group was 52.075 ± 7.82 years, and the difference in age between the two groups was not statistically significant (*p* > 0.05). Treatment: patients were given oral THD 150 mL/dose twice/day for 4 weeks. We collected 5 mL of fasting venous blood from the participants in the morning, rested at 4°C for 1 h, and centrifuged for 10 min (3000 r-min-1). Then, the supernatant was removed and stored frozen at −80 °C. Blood was collected from the patients in pre-treatment (A) and post-treatment (B) and once from the normal group (NC).

### 2.2 Efficacy evaluation

The patient’s self-reported pain was scored pre- and post-treatment using a visual analogue scale (VAS), with higher scores indicating severe subjective pain. The patient’s lumbar spine function was assessed pre- and post-treatment using the Japanese Orthopaedic Association (JOA) Assessment Treatment Score, with higher JOA scores indicating better recovery (Lyu et al., 2021).

### 2.3 Serum testing

Changes in serum interleukin-1-beta (IL-1β), tumour necrosis factor-alpha (TNF-α), and prostaglandin-E2 (PGE2) levels in the two groups of patients pre- and post-treatment were measured according to the method described in the ELISA kit for human use.

### 2.4 Metabolomics analysis

#### 2.4.1 Sample preparation and extraction

The sample stored at −80 °C refrigerator was thawed on ice and vortexed for 10 s 50 μL of sample and 300 μL of 20% acetonitrile methanol internal standard extract were added into a 2 mL microcentrifugetube. The sample was vortexed for 3 min and then centrifuged at 12,000 rpm for 10 min (4 °C). 200 μL of the supernatant was collected and placed in −20 °C for 30 min, and then centrifuged at 12,000 rpm for 3 min (4 °C). A 180 μL aliquots of supernatant were transferred for LC-MS analysis.

#### 2.4.2 UPLC conditions (T3)

All samples were acquired by Waters ACQUITY UPLC combined with the Xevo TQ-S Micro mass spectrometer system followed machine orders. The analytical conditions were as follows, UPLC: column, Waters ACQUITY UPLC HSS T3 C18 (1.8 µm, 2.1 mm*100 mm); column temperature, 40 °C; flow rate, 0.4 mL/min; injection volume, 2μL; solvent system, water (0.1% formic acid): acetonitrile (0.1% formic acid); gradient program, 95:5 V/V at 0 min, 10:90 V/V at 11.0 min, 10:90 V/V at 12.0 min, 95:5 V/V at 12.1 min, 95:5 V/V at 14.0 min.

#### 2.4.3 Sample Quality Control analysis

Quality Control (QC) samples were prepared from a mixture of sample extracts and were used to analyse the reproducibility of the samples under the same processing method. During the instrumental analysis, one QC sample was inserted for every ten samples tested for analysis to monitor the reproducibility of the analytical process.

#### 2.4.4 Differential metabolite and metabolic pathway analysis

The original data file acquisited by LC-MS was converted into mzML format by ProteoWizard software. Peak extraction, peak alignment and retention time correction were respectively performed by XCMS program. The “SVR” method was used to correct the peak area. The peaks with detetion rate lower than 50% in each group of samples were discarded. After that, metabolic identification information was obtained by searching the laboratory’s self-built database, integrated public database, AI database and metDNA.

Principal component analysis (PCA) was performed by statistics function prcomp within R (www.r-project.org). The data was unit variance scaled before unsupervised PCA. For two-group analysis, differential metabolites were determined by VIP (VIP ≥1), *p*-value (*p*-value <0.05, Student’s t test) and absolute Log2FC (|Log2FC| ≥ 1.0). VIP values were extracted from orthogonal partial least squares discriminant analysis (OPLS-DA) result, which also contain score plots and permutation plots, was generated using R package MetaboAnalystR. The data was log transform and mean centering before OPLS-DA. In order to avoid overfitting, a permutation test (200 permutations) was performed. Identified metabolites were annotated using KEGG Compound database (http://www.kegg.jp/kegg/compound/), annotated metabolites were then mapped to KEGG Pathway database (http://www.kegg.jp/kegg/pathway.html). Significantly enriched pathways are identified with a hypergeometric test’s *p*-value for a given list of metabolites.

### 2.5 Statistical analysis

SPSS 24.0 was used to analyse the data. χ2 test was applied to compare the differences between groups, and the *t*-test was used to compare the measurement data at the test level of a = 0.05. All statistical tests were performed using a two-sided test, and *p* < 0.05 would be considered a statistically significant difference.

## 3 Results

### 3.1 Comparison of patients’ VAS and JOA scores pre- and post-treatment

**FIGURE 1:**
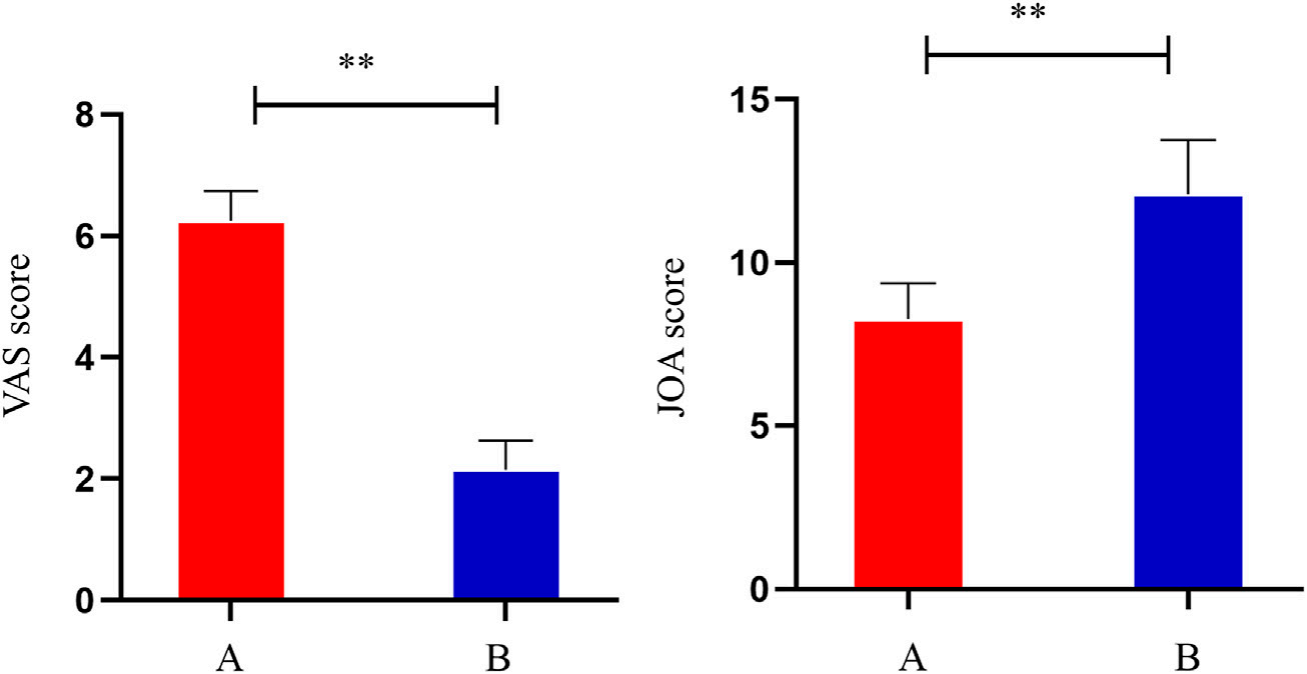
VAS scores and JOA scores of patients before and after treatment. **: *p* < 0.01.

Compared to the pre-treatment period (A), the VAS scores of the patients decreased significantly after treatment (B) (*p* < 0.05), indicating that THD was able to reduce the pain of patients with LSS. Compared to the pre-treatment period (A), the JOA scores of the patients increased significantly after treatment (B) (*p* < 0.05). Thus, the results indicated that THD could improve the function of the lumbar spine in patients with LSS (Figure 1).

### 3.2 Comparison of serum inflammatory factors

**FIGURE 2:**
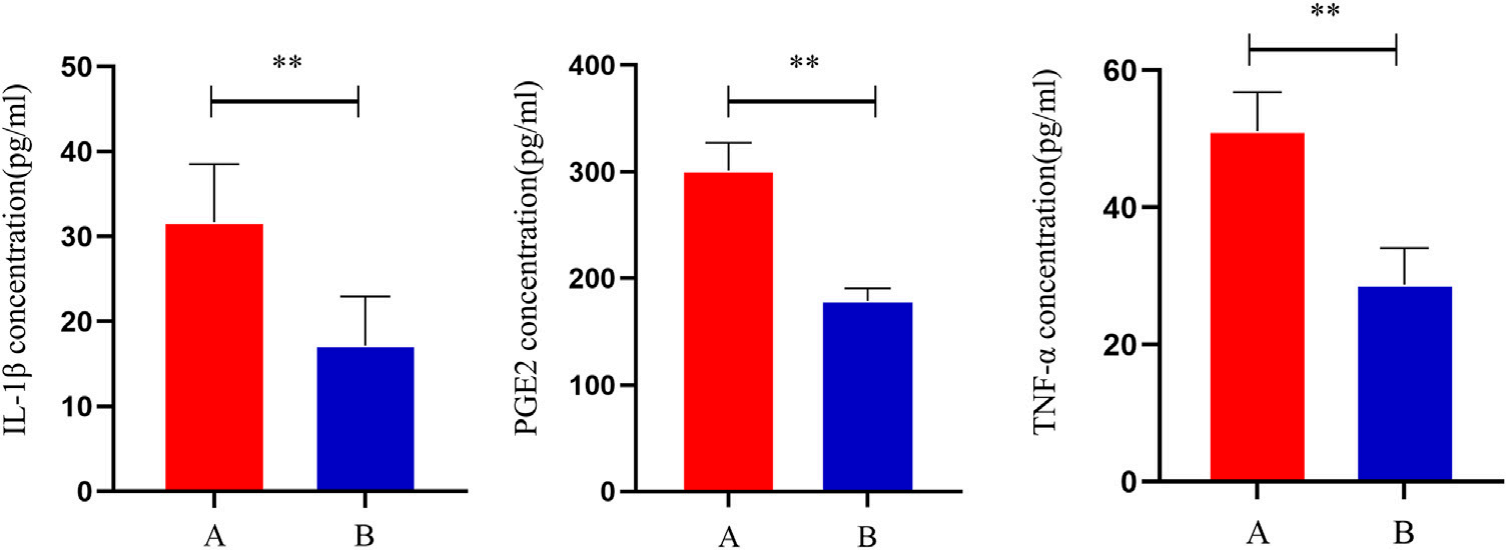
Changes in serum levels of IL-1β, TNF-α and PGE2 in patients before and after treatment. **: *p* < 0.01.

Compared to group A, serum IL-1β, TNF-α, and PGE2 levels were significantly lower in group B (*p* < 0.05), indicating that THD could improve the inflammation level in the serum of LSS patients (Figure 2).

### 3.3 Metabolomic results

#### 3.3.1 Metabolic routine analysis

**FIGURE 3:**
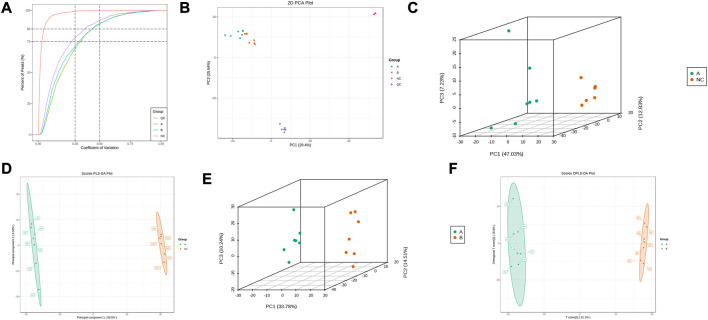
Metabolic Pulmonary Analysis. **(A)** CV distribution of each group of samples; **(B)** PCA score of each group of samples with mass spectral data of QC samples; **(C)** PCA score between A and NC; **(D)** OPLS-DA analysis between A and NC; **(E)** PCA score between A and B; **(F)** OPLS-DA analysis between A and B.

This experiment selected 20 samples and divided them into three groups for metabolic studies. We detected 3412 metabolites. According to the analysis of metabolite composition, the main component was benzene and substituted derivatives, amino acid and its metabolites, heterocyclic compounds, and organic acid and its derivatives (Supplementary Figure S1). Overlap display analyses of total ion flow plots (TIC plots) were analysed using mass spectrometric detection. Different QC samples showed high overlap curves for metabolite detection of total ion flow, i.e., consistent retention times and peak intensities (Supplementary Figure S2), indicating good signal stability of the mass spectrometry for the same sample at different times. The coefficient of variation (CV) is the ratio of the standard deviation of the original data to the mean of the original data and reflects the degree of dispersion of the data. The empirical cumulative distribution function (ECDF) can be used to analyse the frequency of substances with CVs smaller than the reference value. In this study, the percentage of substances with CV values less than 0.3 was higher than 85%, indicating that the experimental data were very stable (Figure 3A).

PCA results showed trends in metabolome separation between groups and whether metabolomes differ within sample groups (Ho et al., 2013). The samples from the different groups in the PCA plot were clustered overall (Figure 3B), indicating good similarity within groups. Furthermore, QC samples were clustered and located in the middle of the other three groups, indicating good system stability, while the ability to separate the three groups indicated significant differences between groups. B lies between A and NC, suggesting that THD improved serum metabolism in patients with LSS. PLS-DA combined orthogonal signal correction and PLS-DA methods, with OPLS-DA maximising group differentiation and facilitating the search for differential metabolites compared to PCA. The characteristics between the NC and A, and A and B groups were well distinguished from each other in both the PCA and OPLS-DA score plots (Figures 3C–F). In the permutation test of OPLS-DA, R2X = 0.518, R2Y = 1, Q2 = 0.987 in NC and A group, R2X = 0.477, R2Y = 0.999, Q2 = 0.945 in A and B group, and *p* < 0.05, indicating that the OPLS-DA model has a high explanatory and predictive rate. In the permutation test of PLS-DA, R2X = 0.677, R2Y = 1, Q2 = 0.997 in NC and group A, R2X = 0.498, R2Y = 0.999, Q2 = 0.99 in A and B group, *p* < 0.05, indicating that the PLS-DA model has a high explanatory and predictive rate. (Supplementary Figure S3).

#### 3.3.2 Screening for differential metabolites

**FIGURE 4:**
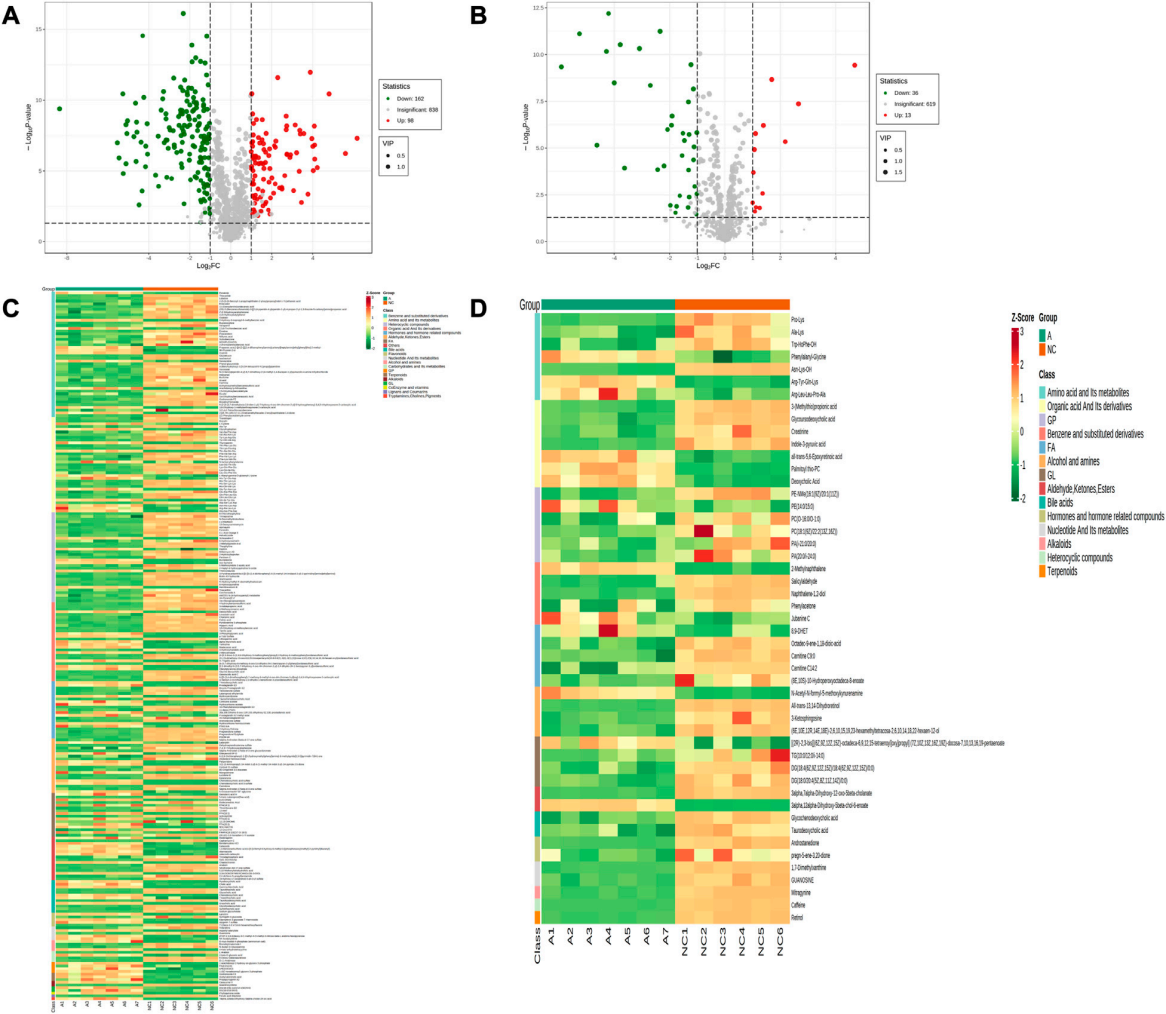
Expression of differential metabolites in NC vs. group A. **(A)** volcano plot of differential metabolites under anionic conditions; **(B)** volcano plot of differential metabolites under cationic conditions; **(C)** heat map of differential metabolite expression between samples under anionic conditions; **(D)** heat map of differential metabolite expression between samples under cationic conditions.

**FIGURE 5:**
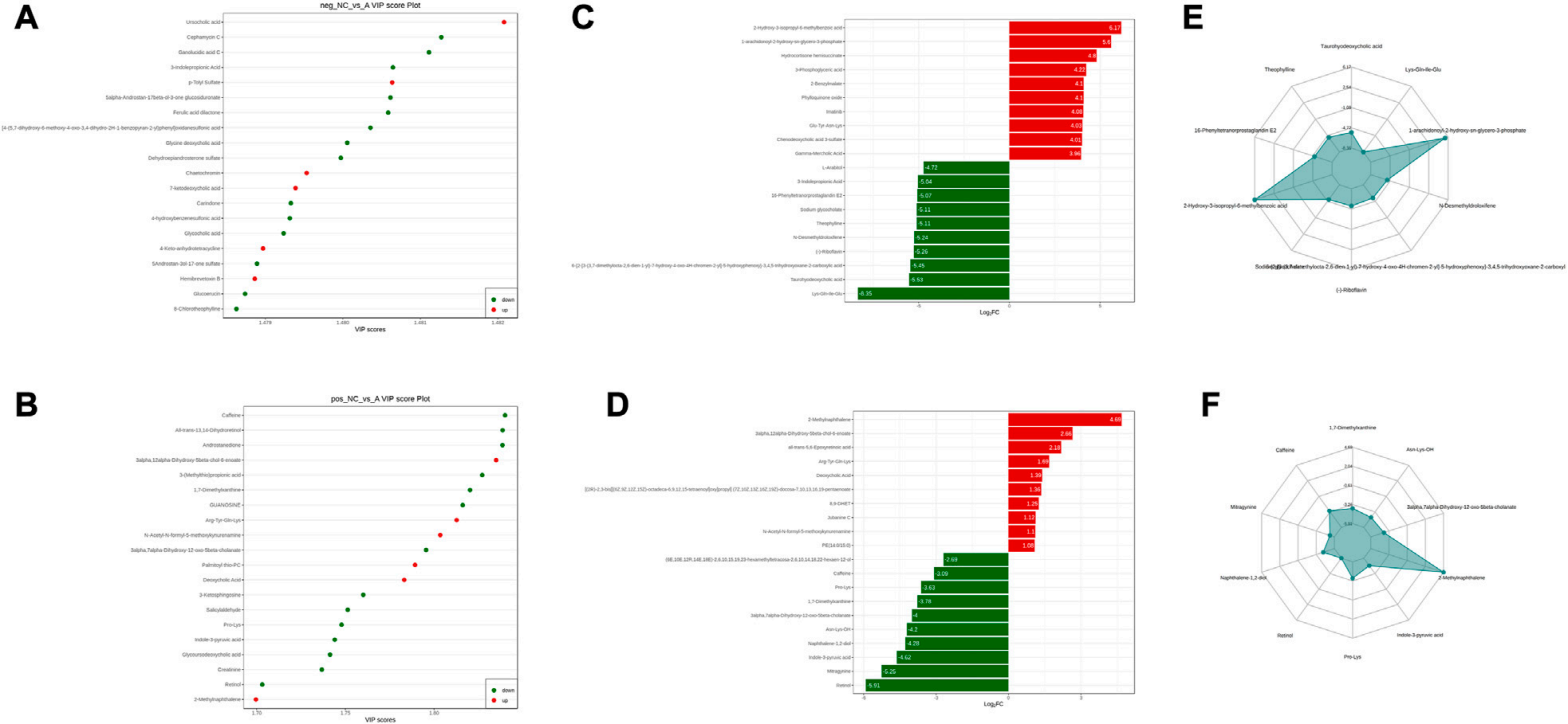
NC vs. group A differential metabolite screening. **(A)** top 20 potential differential metabolites with VIP ≥1 under anionic conditions; **(B)** top 20 potential differential metabolites with VIP ≥1 under cationic conditions; **(C)** top 10 differential metabolites with up- and downregulated ploidy change values under anionic conditions; **(D)** top 10 differential metabolites with up- and downregulated ploidy change values under cationic conditions; **(E)** radar plot under anionic conditions selectively showing ten representative differential metabolites with change fractions; **(F)** radar plot under cationic conditions selectively showing ten representative differential metabolites with change fractions.

Comparing the NC group with group A, there were 309 differential metabolites satisfying VIP >1, |Log2FC| ≥ 1.0 and *p* < 0.05, including 260 under anionic conditions, 98 upregulated and 162 downregulated; and 49 differential metabolites under cationic conditions, including 13 upregulated and 36 downregulated (Figures 4A, B). Moreover, heat maps were used to demonstrate the biomarkers between the groups and the differential expression of the biomarkers between the groups (Figures 4C, D). The top 20 potential biomarkers with VIP ≥1 included ursocholic acid, cephamycin C, ganolucidic acid C, ferulic acid dilactone, glycine deoxycholic acid caffeine, all-trans-13,14-Dihydroretinol, androstanedione, Arg-Tyr-Gln-Lys (Arginine-Tyrosine-Glutamine-Lysine), deoxycholic acid (Figures 5A, B). Based on the fold change values, the top 10 upregulated and downregulated biomarkers in the NC/A group comparison were mainly 2-Hydroxy-3-isopropyl-6-methyl benzoic acid, 3-Phosphoglyceric acid, Lys-Gln-Ile-Glu (lysine-glutamine-isoleucine-glutamic acid), taurohyodeoxycholic acid, 2-Methylnaphthalene 3alpha, 12alpha-Dihydroxy-5beta-chol-6-enoate, retinol, mitragynine (Figures 5C, D). In addition, the radar plot selectively shows ten representative biomarkers with variable scores, including Lys-Gln-Ile-Glu, 2-Hydroxy -3-isopropyl-6-methyl benzoic acid, taurohyodeoxycholic acid, 2-Methylnaphthalene, caffeine, mitragynine (Figures 5E, F). This suggests that amino acid metabolism and lipid metabolism abnormalities might be associated with developing LSS.

**FIGURE 6:**
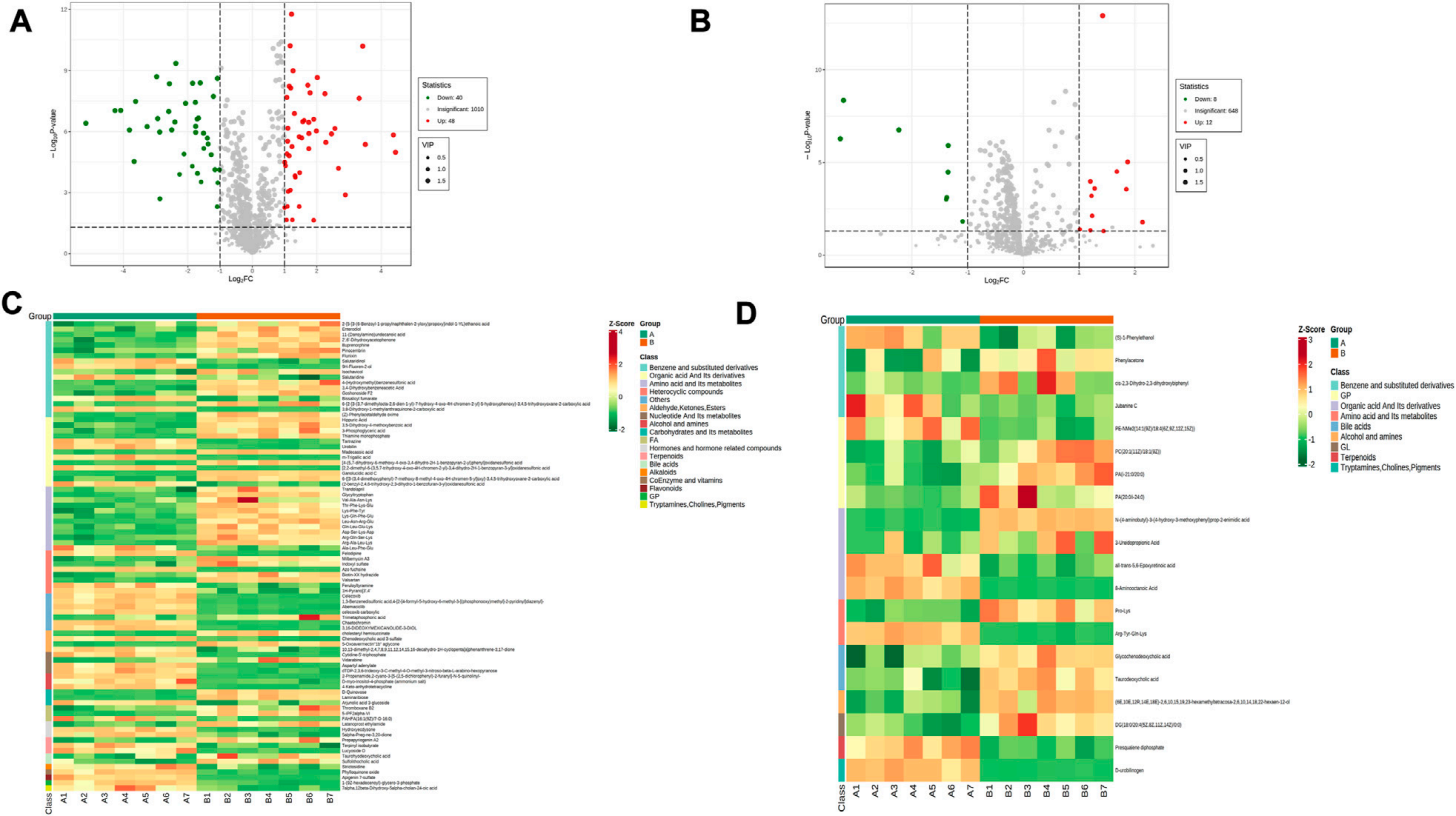
Expression of differential metabolites in groups A vs. B **(A)** volcano plot of differential metabolites under anionic conditions; **(B)** volcano plot of differential metabolites under cationic conditions; **(C)** heat map of differential metabolite expression between samples under anionic conditions; **(D)** heat map of differential metabolite expression between samples under cationic conditions.

**FIGURE 7:**
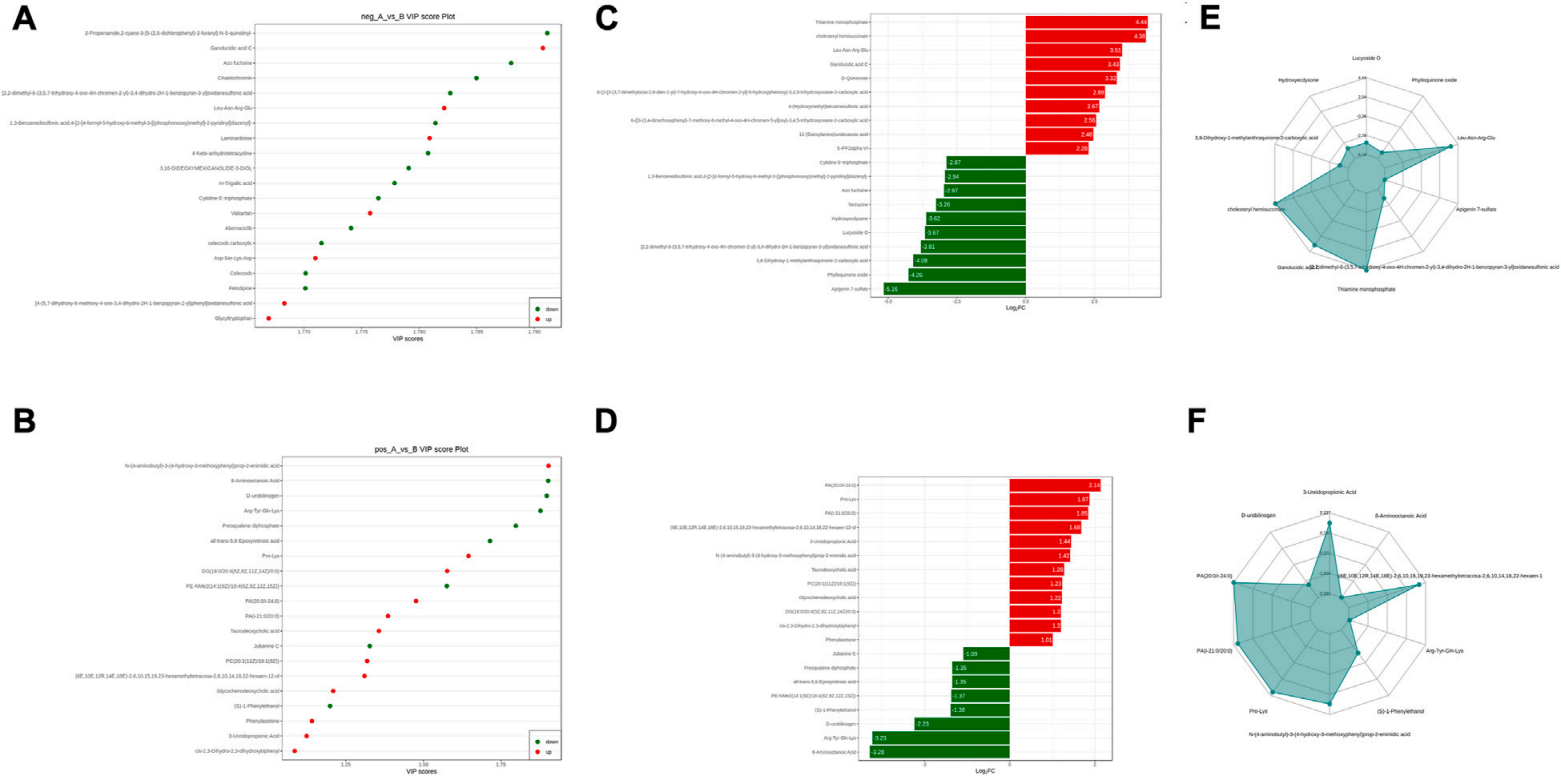
Groups A vs. B differential metabolite screening. **(A)** top 20 potential differential metabolites with VIP ≥1 under anionic conditions; **(B)** top 20 potential differential metabolites with VIP ≥1 under cationic conditions; **(C)** top ten differential metabolites with up- and downregulated ploidy change values under anionic conditions; **(D)** top ten differential metabolites with up- and downregulated ploidy change values under cationic conditions; **(E)** radar plot under anionic conditions selectively showing ten representative differential metabolites with change fractions; **(F)** radar plot under cationic conditions selectively showing ten representative differential metabolites with change fractions.

The sera from groups A and B were subjected to metabolomic analysis, and 309 differential metabolites satisfying VIP >1, Log2FC > 1 and *p* < 0.05, including 88 under anionic conditions, 48 upregulated and 40 downregulated, and 20 differential metabolites under cationic conditions, including 12 upregulated and 8 downregulated (Figures 6A, B). Heat maps were used to demonstrate the biomarkers in each group and differential expression between groups (Figures 6C, D). Of these, the top 20 potential biomarkers with VIP ≥1 were mainly ganolucidic acid C, chaetochromin, Leu-Asn-Arg-Glu, laminaribiose, N-(4-aminobutyl)-3-(4-hydroxy-3-methoxyphenyl) prop-2-enimidic acid, 8-Aminooctanoic Acid, D-urobilinogen, Arg-Tyr-Gln-Lys (Figures 7A, B). The top ten upregulated and downregulated biomarkers in the A/B group comparison, based on the fold change values, contained Thiamine monophosphate, cholesteryl hemisuccinate, Leu-Asn-Arg-Glu (Leucine-Asparagine-Arginine- Glutamic acid), Ganolucidic acid C, D-Quinovose, 8-Aminooctanoic Acid, Arg-Tyr-Gln-Lys, D-urobilinogen, (S)-1-Phenylethanol, all-trans-5,6- Epoxyretinoic acid (Figures 7C, D). In addition, the radar plot selectively shows ten representative biomarkers with variable fractions of Leu-Asn-Arg-Glu, Phylloquinone oxide, Hydroxyecdysone, Arg-Tyr-Gln-Lys, D urobilinogen, and (S)-1-Phenylethanol (Figures 7E, F). The results suggested that THD could regulate amino acid and lipid metabolisms in the body of LSS patients, thus exerting its therapeutic effects.

Furthermore, Venn diagrams were used to identify the intersection between NC/A and A/B and identified 76 common biomarkers. Among those, 41 had significantly different levels in the normal group compared to pre-treatment and were significantly restored after THD treatment (Table 1). Most of those biomarkers belonged to amino acids and their metabolites, bile acids, organic acids and their derivatives, indicating that THD could regulate amino acid and lipid metabolisms in LSS patients.

**TABLE 1:** Levels of 41 biomarkers were significantly restored by THD treatment.

	Formula	Compounds	Class I	VIP	*p*-value	Log2FC	Type
MW0109398	C11H21N3O3	Pro-Lys	Amino acid and Its metabolites	1.65	9.35E-06	1.87	up
MW0053750	C26H43NO5	Glycochenodeoxycholic acid	Bile acids	1.21	6.31E-04	1.22	up
MW0139070	C15H20O4	N-(4-aminobutyl)-3-(4-hydroxy-3-methoxyphenyl)prop-2-enimidic acid	Organic acid And Its derivatives	1.90	1.25E-13	1.42	up
MEDN0841	C4H8N2O3	3-Ureidopropionic Acid	Organic acid And Its derivatives	1.12	5.00E-02	1.44	up
MEDP1233	C33H42N4O6	D-urobilinogen	Tryptamines, Cholines,Pigments	1.90	1.76E-07	−2.23	down
MW0063749	C26H45NO6S	Taurodeoxycholic acid	Bile acids	1.36	2.51E-04	1.28	up
MW0140638	C8H10O	(S)-1-Phenylethanol	Benzene and substituted derivatives	1.20	9.83E-04	−1.38	down
MW0015647	C20H28O3	all-trans-5,6-Epoxyretinoic acid	Organic acid And Its derivatives	1.71	3.30E-05	−1.35	down
MW0155485	C9H10O	Phenylacetone	Benzene and substituted derivatives	1.14	4.03E-02	1.01	up
mws3144_N	C8H17NO2	8-Aminooctanoic Acid	Organic acid And Its derivatives	1.90	5.26E-07	−3.28	down
MW0147534	C12H12O2	cis-2,3-Dihydro-2,3-dihydroxybiphenyl	Benzene and substituted derivatives	1.09	4.51E-02	1.20	up
MW0155740	C30H52O7P2	Presqualene diphosphate	Terpenoids	1.80	1.21E-06	−1.35	down
MW0060196	C39H68NO8P	PE-NMe2(14:1(9Z)/18:4(6Z,9Z,12Z,15Z))	GP	1.58	7.68E-04	−1.37	down
MW0057219	C46H88NO8P	PC(20:1(11Z)/18:1(9Z))	GP	1.32	7.52E-03	1.23	up
MW0056644	C44H87O8P	PA(i-21:0/20:0)	GP	1.39	2.78E-04	1.85	up
MW0055921	C47H93O8P	PA(20:0/i-24:0)	GP	1.48	1.65E-02	2.14	up
MW0107606	C39H47N5O5	Jubanine C	Benzene and substituted derivatives	1.33	1.51E-02	−1.09	down
MW0050034	C41H72O5	DG(18:0/20:4(5Z,8Z,11Z,14Z)/0:0)	GL	1.58	1.05E-04	1.20	up
MW0145540	C26H43N9O7	Arg-Tyr-Gln-Lys	Amino acid and Its metabolites	1.88	4.48E-09	−3.23	down
MW0166423	C30H50O	(6E,10E,12R,14E,18E)-2,6,10,15,19,23-hexamethyltetracosa-2,6,10,14,18,22-hexaen-12-ol	Alcohol and amines	1.31	3.10E-05	1.68	up

#### 3.3.3 Metabolite pathway analysis

**FIGURE 8:**
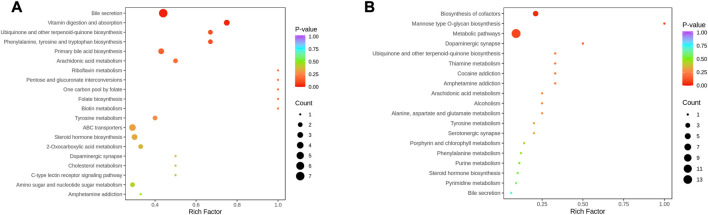
KEGG Enrichment analysis of differential metabolites. **(A)**: NC vs. A; **(B)** A vs. B; the dot’s colour is the *p*-value. The redder it is, the more significant the enrichment is. The dot size represents the number of differential metabolites in the pathway enriched).

Metabolites interact with the organism to form different pathways. Metabolic signalling in KEGG showed that bile secretion, vitamin digestion and absorption, ubiquinone and other terpenoid-quinone biosynthesis, primary bile acid biosynthesis, phenylalanine, tyrosine and tryptophan biosynthesis were altered compared to NC (Figure 8A). In contrast, metabolic pathways, biosynthesis of cofactors, and mannose-type O-glycan biosynthesis were regulated in the serum of patients before and after THD treatment, which might be an effective pathway for THD treatment of LSS (Figure 8B).

**FIGURE 9:**
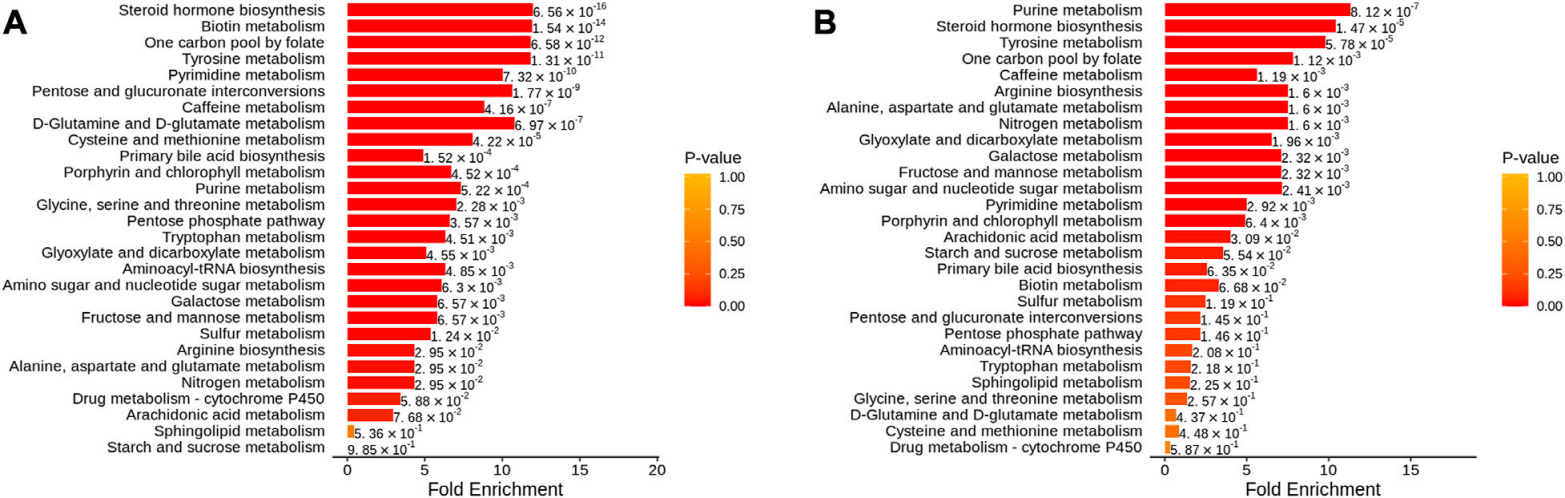
MSEA of differential metabolites. **(A)**: NC vs. A; **(B)** A vs. B; The dot’s colour represents *p*-value).

In addition, metabolic enrichment analysis (MSEA) does not require the specification of clear thresholds for differential metabolites and identifies significantly different metabolomes through a series of metabolic ensembles, each representing a biological function (Ma et al., 2022). The results showed that steroid hormone biosynthesis, biotin metabolism, one carbon pool by folate, tyrosine metabolism, and pyrimidine metabolism were the main pathways of metabolic differences between A and NC (Figure 9A). Purine metabolism, steroid hormone biosynthesis, tyrosine metabolism, one carbon pool by folate, and arginine biosynthesis had significant differences before and after THD treatment (groups A and B) (Figure 9B). This suggested a clear overlap between the therapeutic process of THD with LSS and its development. THD can potentially correct amino acid metabolism in patients with LSS and thus achieve therapeutic effects.

## 4 Discussion

Pain is the main symptom and reason for patients to seek medical attention, and low back pain is one of the common symptoms among LSS patients. Unbearable pain and severe functional impairment are the most common reasons patients with LSS undergo spinal surgery (Lurie and Tomkins-Lane, 2016). Treatment of THD is effective in relieving pain and improving the functional movement of the lumbar spine in patients with LSS. Moreover, the effective relief of pain is thought to be closely related to reducing local tissue inflammation in patients(Conaghan et al., 2019). In this study, serum levels of IL-1β, TNF-α and PGE2 decreased significantly after THD treatment compared with those before treatment. In contrast, levels of the pro-inflammatory cytokines IL-1β, TNF-α, and PGE2 were significantly elevated in the serum of surgically induced LSS rats, and it was noted that these inflammatory factors were closely related to pain (Park et al., 2019). Inhibition of the expression of key inflammatory mediators reduces LSS-induced chronic mechanical abnormalities in pain (Lee et al., 2019).

Activation of inflammation contributes to fibrosis and hypertrophy of the ligamentum flavum in the LSS (Sun et al., 2018). Macrophage migration inhibitory factor (MIF) promotes ligamentum flavum proliferation through the Src kinase signaling pathway and extracellular matrix changes through its pro-inflammatory effects. MIF and its mediated inflammatory response are the drivers of ligamentum flavum hypertrophy (Lu et al., 2022). The MIF content is positively correlated with the thickness of the ligamentum flavum, and it promotes fibroblast proliferation and collagen fibrillation which might be an important part of the ligamentum flavum scar repair leading to its hypertrophy. The inflammatory response, which includes both inflammatory damage and repair, is one of the main components of the blood stasis theory in TCM, and herbs with blood activation and blood stasis removal effects can effectively block the inflammatory and pro-proliferative effects of MIF on fibroblasts (Lu, 2020). Modern pharmacological studies have shown that tonifying the kidney and invigorating the blood can effectively reduce the expression of inflammatory factors and inhibit the degeneration of articular cartilage (Wang et al., 2022). THD is a clinically representative formula for tonifying the kidneys and invigorating the blood. Astragalus polysaccharide, the active ingredient of Huang Qi, can inhibit macrophage migration inhibitory factor(Liao et al., 2020). In addition, Huang Qi, Dang Gui and Dan Shen are involved in enhancing angiogenesis and osteogenesis in THD. Their positive effect on bone formation may be related to their ability to promote angiogenesis by acting on substances such as VEGF (Yang et al., 2014). Huang Qi and Dan Shen have been shown to promote the proliferation of bone marrow mesenchymal stem cells and TGF-β1-induced bone marrow mesenchymal stem cells *in vitro*(Cai et al., 2015). Tanshinone IIA is one of the main active phytochemicals isolated from Dan Shen, which has been reported to inhibit osteoclast differentiation and bone resorption by disrupting the actin ring, thereby inhibiting osteoclast formation and bone erosion(Kwak et al., 2008; Nicolin et al., 2010).

Based on the apparent clinical effects of THD on LSS, we explored its mechanism of action using metabolomics and showed that the levels of 41 potential metabolites were significantly restored by THD treatment, including Chenodeoxycholic acid 3-sulfate, Taurohyodeoxycholic acid, 3,5-Dihydroxy-4-methoxy benzoic acid, Pinocembrin. Bile acids have multiple biological functions and are involved in pathways, including lipid and glucose metabolism, energy expenditure and inflammation, thereby regulating metabolism-related diseases. By quantifying circulating levels of specific bile acids, Taurohyodeoxycholic acid was found to be negatively associated with diabetes(Choucair et al., 2020). The association between diabetes and LSS is a risk factor for developing LSS, and prolonged and poorly controlled hyperglycaemia can exacerbate disc degeneration(Asadian et al., 2016; Kakadiya et al., 2020). Upregulation of Chenodeoxycholic acid 3-sulfate levels is thought to disrupt the metabolic processes of the body’s antioxidant defence (Wang et al., 2021). Similarly, 3,5-Dihydroxy-4-methoxybenzoic acid, a new antioxidant flavonoid, can effectively inhibit the level of reactive oxygen species and regulate the inflammatory process(Oladele Oladimeji et al., 2018; Gadallah et al., 2020). Pinocembrin attenuates glucocorticoid-induced apoptosis in osteoblasts by inhibiting the PI3K/Akt/mTOR pathway to activate autophagy and may have a protective effect on osteocytes(Wang et al., 2020). The levels of these flavonoid molecules were significantly higher post-treatment compared to pre-treatment. Thus, THD might exert its anti-inflammatory mechanism by regulating the levels of flavonoid molecule metabolites.

The analysis of the different metabolites between patients in the NC group and group A found that it was mainly steroid hormone biosynthesis. Biotin metabolism, one carbon pool by Folate, Tyrosine metabolism, Pyrimidine metabolism and other pathways were related, while THD was related mainly through Purine Metabolism. Steroid hormone biosynthesis, Tyrosine metabolism, one carbon pool by Folate, Arginine biosynthesis, and other pathways regulate body metabolism and play a therapeutic role. A study using transcriptomic data found that purine metabolism significantly affected gene expression in patients with ligamentum flavum ossification, with xanthine dehydrogenase being a key regulator (Li et al., 2020). Whereas ligamentum flavum fibrosis and ossification be the primary pathology of ligamentum flavum hypertrophy, ligamentum flavum hypertrophy is the most crucial component of LSS (Yamada et al., 2021). The intervention of steroid hormone biosynthesis and amino acid metabolic pathways can effectively prevent bone loss (Xia et al., 2019). Epidural steroid injections improve serum monocyte chemotactic protein-1, biomarkers of nerve root injury and electromyography in patients with LSS (Lin et al., 2020).

## 5 Conclusion

**FIGURE 10:**
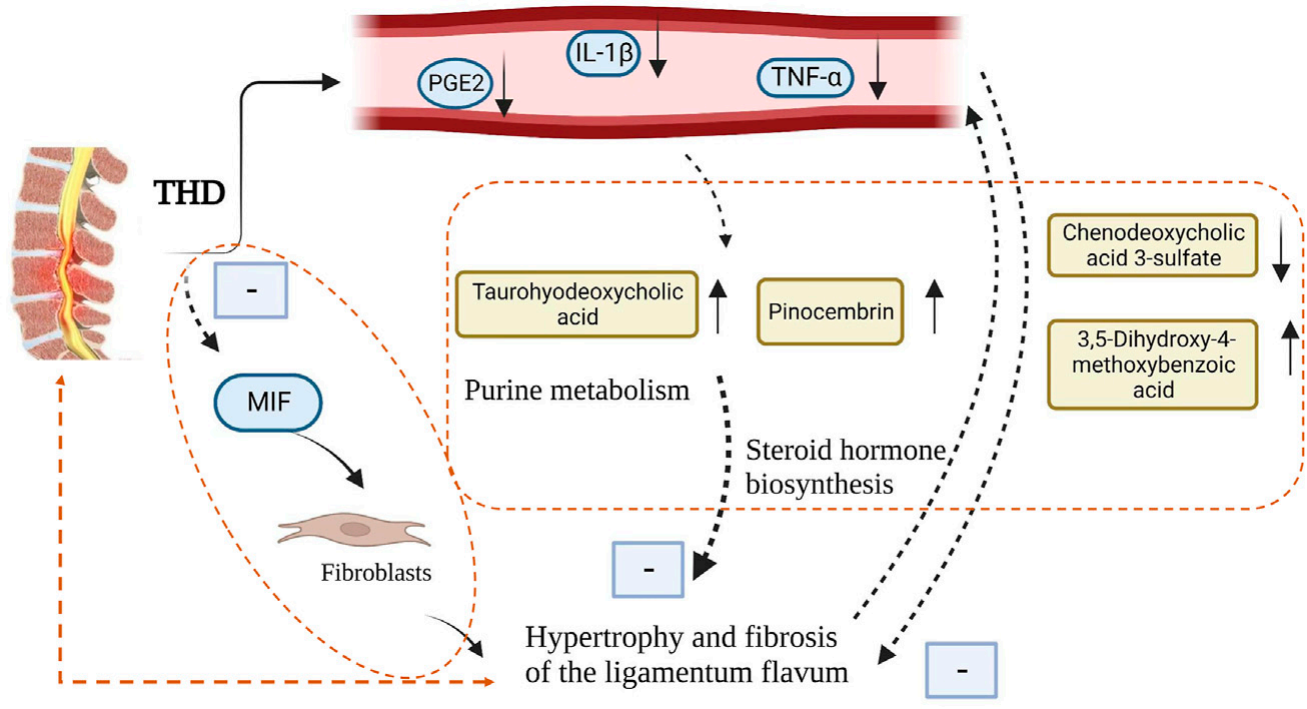
THD intervenes in LSS metabolic pathway mechanism map.

THD can effectively improve pain and lumbar spine function in patients with LSS and reduce serum levels of IL-1β, TNF-α and PGE2-related inflammatory factors in patients. Its mechanism of action might be related to reducing the inflammatory response, improving amino acid metabolism and lipid metabolism, and relieving ligamentum flavum hypertrophy and fibrosis (Figure 10).

## Data Availability

The raw data supporting the conclusion of this article will be made available by the authors, without undue reservation.

## Supplementary material

SUPPLEMENTARY FIGURE S1 Metabolite Class Composition Ring Diagram. (Note: Each colour represents a metabolite category and the area of the colour block indicates the proportion of that category).

SUPPLEMENTARY FIGURE S2 TIC overlay for QC sample mass spectrometry.
