# Supplementary figures and images for: Modulation of the biological network of lumbar spinal stenosis by Tongdu Huoxue Decoction based on clinical metabolomics
Source: Front Mol Biosci. 2023 Mar 21;10:1074500. doi: 10.3389/fmolb.2023.1074500 (PMC10070985; doi:10.3389/fmolb.2023.1074500)

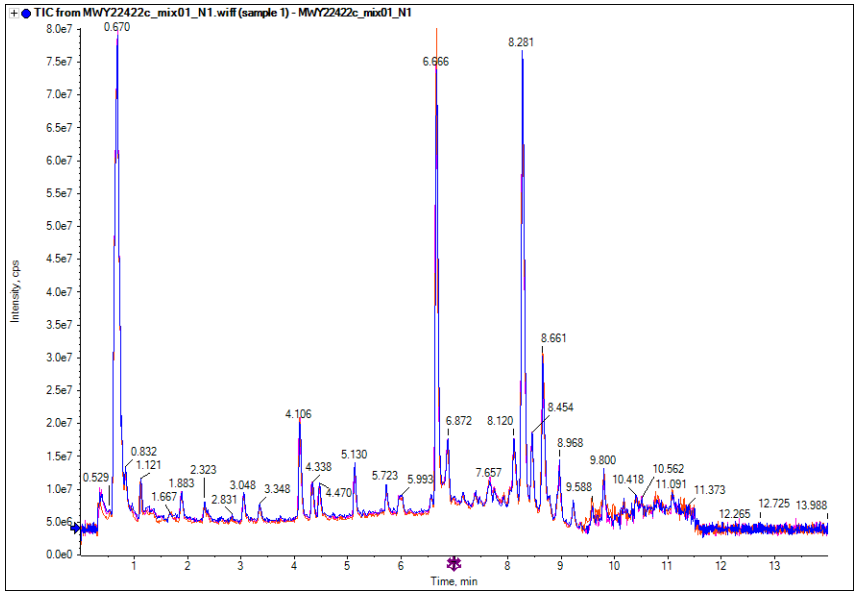

Supplement: Supplementary file 2 [file Image2.PNG]
